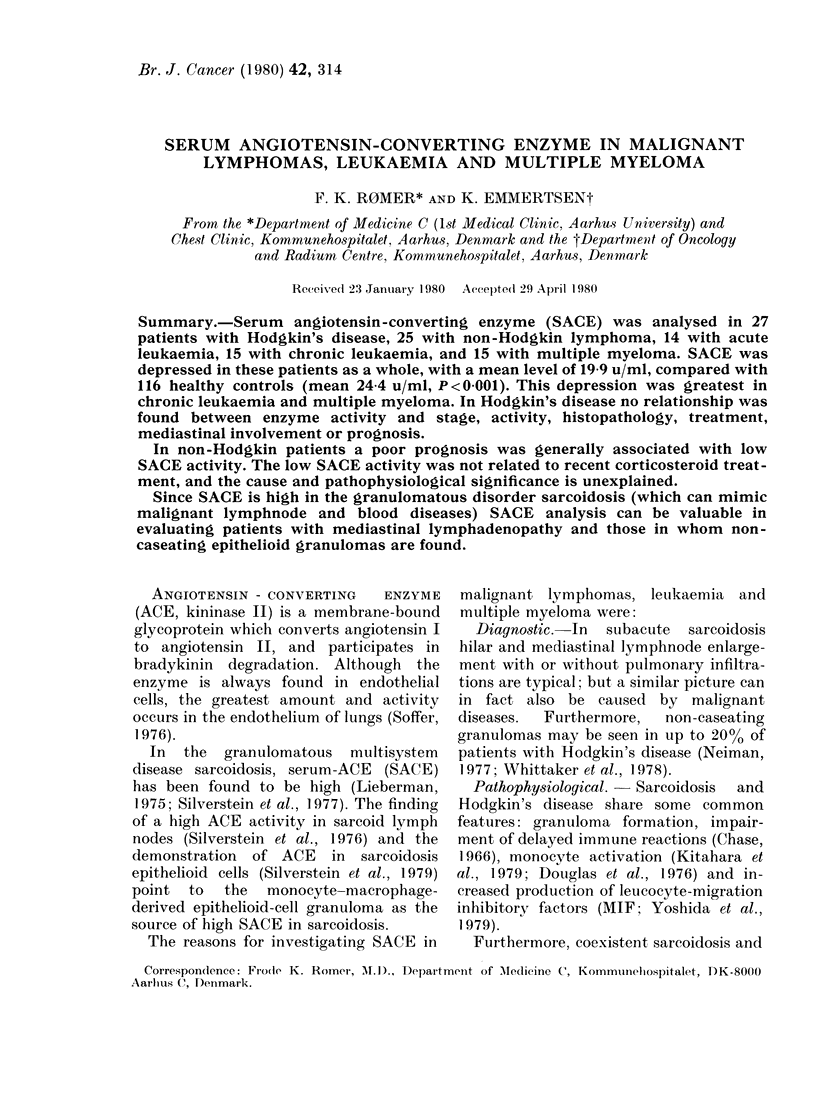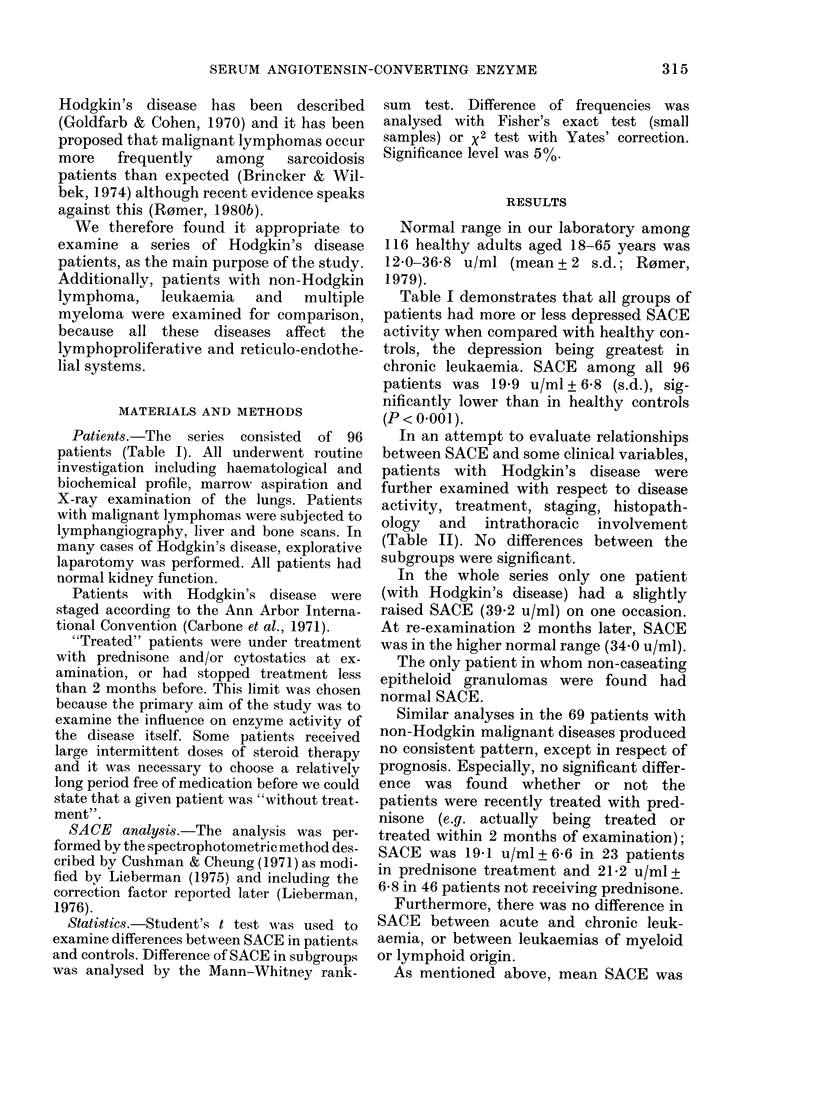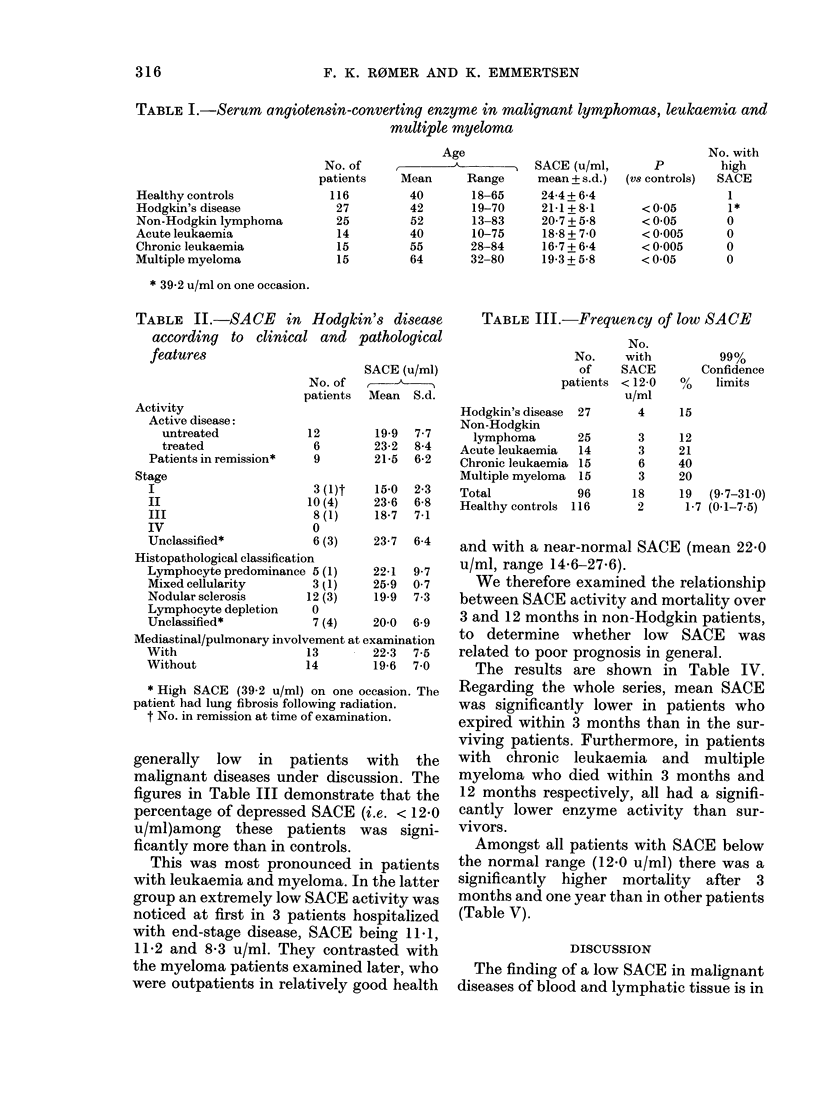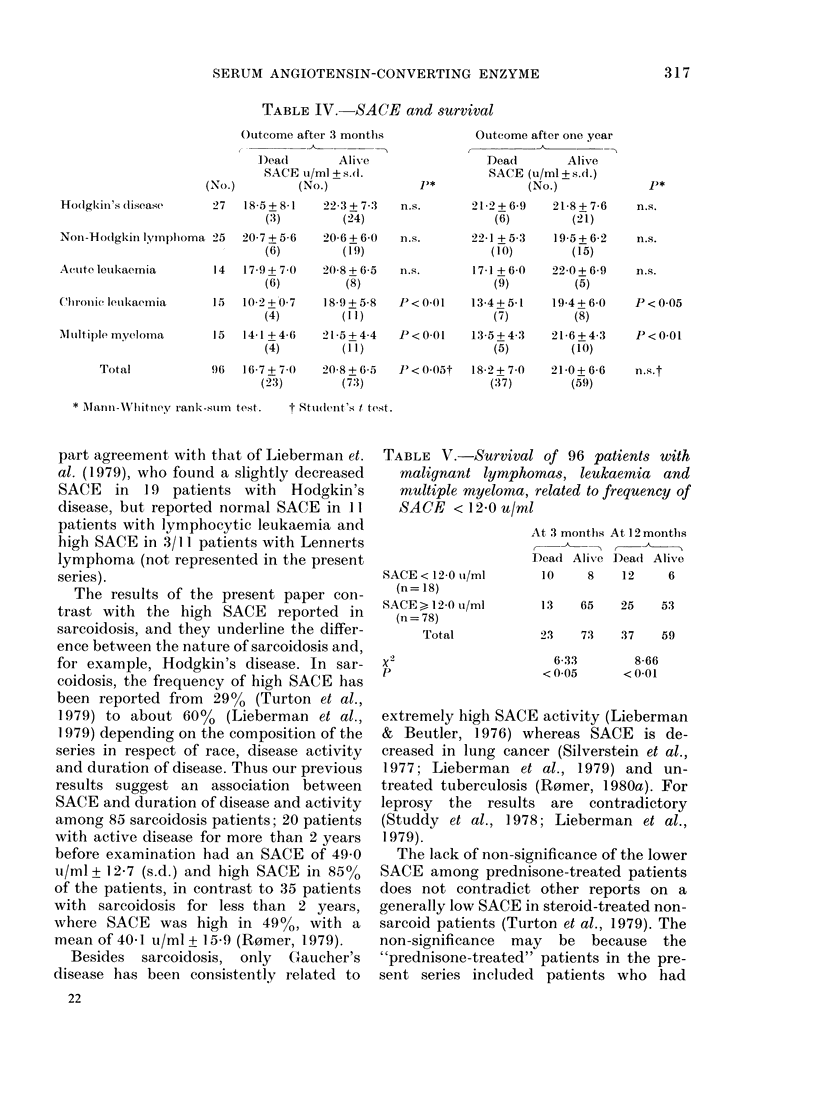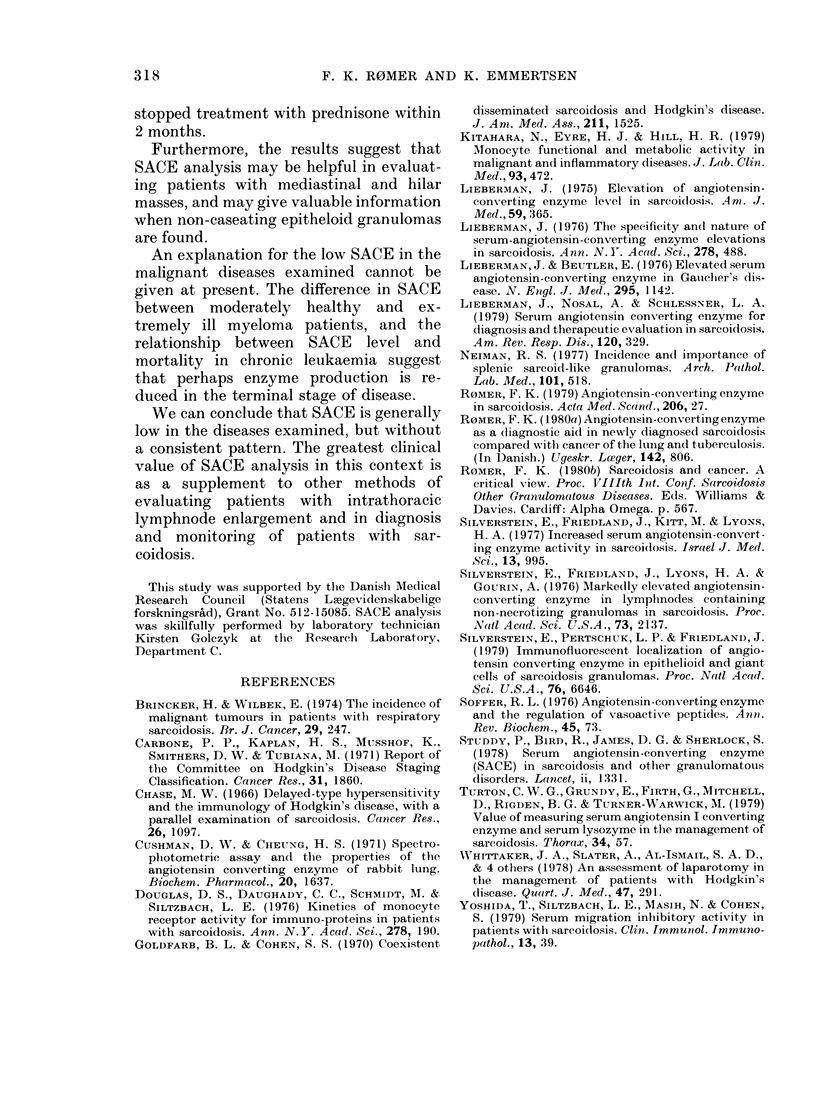# Serum angiotensin-converting enzyme in malignant lymphomas, leukaemia and multiple myeloma.

**DOI:** 10.1038/bjc.1980.232

**Published:** 1980-08

**Authors:** F. K. Rømer, K. Emmertsen

## Abstract

Serum angiotensin-converting enzyme (SACE) was analysed in 27 patients with Hodgkin's disease, 25 with non-Hodgkin lymphoma, 14 with acute leukaemia, 15 with chronic leukaemia, and 15 with multiple myeloma. SACE was depressed in these patients as a whole, with a mean level of 19.9 mu/ml, compared with 116 healthy controls (mean 24.4 mu/ml, P < 0.001). This depression was greatest in chronic leukaemia and multiple myeloma. In Hodgkin's disease no relationship was found between enzyme activity and stage, activity, histopathology, treatment, mediastinal involvement or prognosis. In non-Hodgkin patients a poor prognosis was generally associated with low SACE activity. The low SACE activity was not related to recent corticosteroid treatment, and the cause and pathophysiological significance is unexplained. Since SACE is high in the granulomatous disorder sarcoidosis (which can mimic malignant lymphnode and blood diseases) SACE analysis can be valuable in evaluating patients with mediastinal lymphadenopathy and those in whom non-caseating epitheliod granulomas are found.


*Br. J. Cancer* (1980) **42**, 314

### SERUM ANGIOTENSIN-CONVERTING ENZYME IN MALIGNANT LYMPHOMAS, LEUKAEMIA AND MULTIPLE MYELOMA

F. K. RØMER* AND K. EMMERTSEN†

*From the *Department of Medicine C (1st Medical Clinic, Aarhus University) and Chest Clinic, Kommunehospitalet, Aarhus, Denmark and the †Department of Oncology and Radium Centre, Kommunehospitalet, Aarhus, Denmark*

Received 23 January 1980 Accepted 29 April 1980

**Summary.—Serum angiotensin-converting enzyme (SACE) was analysed in 27 patients with Hodgkin's disease, 25 with non-Hodgkin lymphoma, 14 with acute leukaemia, 15 with chronic leukaemia, and 15 with multiple myeloma. SACE was depressed in these patients as a whole, with a mean level of 19·9 u/ml, compared with 116 healthy controls (mean 24·4 u/ml, $P < 0{\cdot}001$). This depression was greatest in chronic leukaemia and multiple myeloma. In Hodgkin's disease no relationship was found between enzyme activity and stage, activity, histopathology, treatment, mediastinal involvement or prognosis.**

**In non-Hodgkin patients a poor prognosis was generally associated with low SACE activity. The low SACE activity was not related to recent corticosteroid treatment, and the cause and pathophysiological significance is unexplained.**

**Since SACE is high in the granulomatous disorder sarcoidosis (which can mimic malignant lymphnode and blood diseases) SACE analysis can be valuable in evaluating patients with mediastinal lymphadenopathy and those in whom non-caseating epithelioid granulomas are found.**

ANGIOTENSIN - CONVERTING ENZYME (ACE, kininase II) is a membrane-bound glycoprotein which converts angiotensin I to angiotensin II, and participates in bradykinin degradation. Although the enzyme is always found in endothelial cells, the greatest amount and activity occurs in the endothelium of lungs (Soffer, 1976).

In the granulomatous multisystem disease sarcoidosis, serum-ACE (SACE) has been found to be high (Lieberman, 1975; Silverstein *et al.*, 1977). The finding of a high ACE activity in sarcoid lymph nodes (Silverstein *et al.*, 1976) and the demonstration of ACE in sarcoidosis epithelioid cells (Silverstein *et al.*, 1979) point to the monocyte–macrophage-derived epithelioid-cell granuloma as the source of high SACE in sarcoidosis.

The reasons for investigating SACE in malignant lymphomas, leukaemia and multiple myeloma were:

*Diagnostic.*—In subacute sarcoidosis hilar and mediastinal lymphnode enlargement with or without pulmonary infiltrations are typical; but a similar picture can in fact also be caused by malignant diseases. Furthermore, non-caseating granulomas may be seen in up to 20% of patients with Hodgkin's disease (Neiman, 1977; Whittaker *et al.*, 1978).

*Pathophysiological.* — Sarcoidosis and Hodgkin's disease share some common features: granuloma formation, impairment of delayed immune reactions (Chase, 1966), monocyte activation (Kitahara *et al.*, 1979; Douglas *et al.*, 1976) and increased production of leucocyte-migration inhibitory factors (MIF; Yoshida *et al.*, 1979).

Furthermore, coexistent sarcoidosis and

Correspondence: Frode K. Romer, M.D., Department of Medicine C, Kommunehospitalet, DK-8000 Aarhus C, Denmark.

Hodgkin's disease has been described (Goldfarb & Cohen, 1970) and it has been proposed that malignant lymphomas occur more frequently among sarcoidosis patients than expected (Brincker & Wilbek, 1974) although recent evidence speaks against this (Rømer, 1980*b*).

We therefore found it appropriate to examine a series of Hodgkin's disease patients, as the main purpose of the study. Additionally, patients with non-Hodgkin lymphoma, leukaemia and multiple myeloma were examined for comparison, because all these diseases affect the lymphoproliferative and reticulo-endothelial systems.

### MATERIALS AND METHODS

*Patients*.—The series consisted of 96 patients (Table I). All underwent routine investigation including haematological and biochemical profile, marrow aspiration and X-ray examination of the lungs. Patients with malignant lymphomas were subjected to lymphangiography, liver and bone scans. In many cases of Hodgkin's disease, explorative laparotomy was performed. All patients had normal kidney function.

Patients with Hodgkin's disease were staged according to the Ann Arbor International Convention (Carbone *et al*., 1971).

"Treated" patients were under treatment with prednisone and/or cytostatics at examination, or had stopped treatment less than 2 months before. This limit was chosen because the primary aim of the study was to examine the influence on enzyme activity of the disease itself. Some patients received large intermittent doses of steroid therapy and it was necessary to choose a relatively long period free of medication before we could state that a given patient was "without treatment".

*SACE analysis*.—The analysis was performed by the spectrophotometric method described by Cushman & Cheung (1971) as modified by Lieberman (1975) and including the correction factor reported later (Lieberman, 1976).

*Statistics*.—Student's *t* test was used to examine differences between SACE in patients and controls. Difference of SACE in subgroups was analysed by the Mann–Whitney rank-sum test. Difference of frequencies was analysed with Fisher's exact test (small samples) or $\chi^2$ test with Yates' correction. Significance level was 5%.

### RESULTS

Normal range in our laboratory among 116 healthy adults aged 18–65 years was 12·0–36·8 u/ml (mean ± 2 s.d.; Rømer, 1979).

Table I demonstrates that all groups of patients had more or less depressed SACE activity when compared with healthy controls, the depression being greatest in chronic leukaemia. SACE among all 96 patients was 19·9 u/ml ± 6·8 (s.d.), significantly lower than in healthy controls ($P < 0{\cdot}001$).

In an attempt to evaluate relationships between SACE and some clinical variables, patients with Hodgkin's disease were further examined with respect to disease activity, treatment, staging, histopathology and intrathoracic involvement (Table II). No differences between the subgroups were significant.

In the whole series only one patient (with Hodgkin's disease) had a slightly raised SACE (39·2 u/ml) on one occasion. At re-examination 2 months later, SACE was in the higher normal range (34·0 u/ml).

The only patient in whom non-caseating epitheloid granulomas were found had normal SACE.

Similar analyses in the 69 patients with non-Hodgkin malignant diseases produced no consistent pattern, except in respect of prognosis. Especially, no significant difference was found whether or not the patients were recently treated with prednisone (*e.g.* actually being treated or treated within 2 months of examination); SACE was 19·1 u/ml ± 6·6 in 23 patients in prednisone treatment and 21·2 u/ml ± 6·8 in 46 patients not receiving prednisone.

Furthermore, there was no difference in SACE between acute and chronic leukaemia, or between leukaemias of myeloid or lymphoid origin.

As mentioned above, mean SACE was

TABLE I.—*Serum angiotensin-converting enzyme in malignant lymphomas, leukaemia and multiple myeloma*

| | No. of patients | Age: Mean | Age: Range | SACE (u/ml, mean ± s.d.) | *P* (*vs* controls) | No. with high SACE |
|---|---|---|---|---|---|---|
| Healthy controls | 116 | 40 | 18–65 | 24·4 ± 6·4 | | 1 |
| Hodgkin's disease | 27 | 42 | 19–70 | 21·1 ± 8·1 | < 0·05 | 1* |
| Non-Hodgkin lymphoma | 25 | 52 | 13–83 | 20·7 ± 5·8 | < 0·05 | 0 |
| Acute leukaemia | 14 | 40 | 10–75 | 18·8 ± 7·0 | < 0·005 | 0 |
| Chronic leukaemia | 15 | 55 | 28–84 | 16·7 ± 6·4 | < 0·005 | 0 |
| Multiple myeloma | 15 | 64 | 32–80 | 19·3 ± 5·8 | < 0·05 | 0 |

* 39·2 u/ml on one occasion.

TABLE II.—*SACE in Hodgkin's disease according to clinical and pathological features*

| | No. of patients | SACE (u/ml) Mean | SACE (u/ml) S.d. |
|---|---|---|---|
| Activity | | | |
| Active disease: | | | |
| untreated | 12 | 19·9 | 7·7 |
| treated | 6 | 23·2 | 8·4 |
| Patients in remission* | 9 | 21·5 | 6·2 |
| Stage | | | |
| I | 3 (1)† | 15·0 | 2·3 |
| II | 10 (4) | 23·6 | 6·8 |
| III | 8 (1) | 18·7 | 7·1 |
| IV | 0 | | |
| Unclassified* | 6 (3) | 23·7 | 6·4 |
| Histopathological classification | | | |
| Lymphocyte predominance | 5 (1) | 22·1 | 9·7 |
| Mixed cellularity | 3 (1) | 25·9 | 0·7 |
| Nodular sclerosis | 12 (3) | 19·9 | 7·3 |
| Lymphocyte depletion | 0 | | |
| Unclassified* | 7 (4) | 20·0 | 6·9 |
| Mediastinal/pulmonary involvement at examination | | | |
| With | 13 | 22·3 | 7·5 |
| Without | 14 | 19·6 | 7·0 |

* High SACE (39·2 u/ml) on one occasion. The patient had lung fibrosis following radiation.
† No. in remission at time of examination.

generally low in patients with the malignant diseases under discussion. The figures in Table III demonstrate that the percentage of depressed SACE (*i.e.* < 12·0 u/ml)among these patients was significantly more than in controls.

This was most pronounced in patients with leukaemia and myeloma. In the latter group an extremely low SACE activity was noticed at first in 3 patients hospitalized with end-stage disease, SACE being 11·1, 11·2 and 8·3 u/ml. They contrasted with the myeloma patients examined later, who were outpatients in relatively good health

TABLE III.—*Frequency of low SACE*

| | No. of patients | No. with SACE < 12·0 u/ml | % | 99% Confidence limits |
|---|---|---|---|---|
| Hodgkin's disease | 27 | 4 | 15 | |
| Non-Hodgkin lymphoma | 25 | 3 | 12 | |
| Acute leukaemia | 14 | 3 | 21 | |
| Chronic leukaemia | 15 | 6 | 40 | |
| Multiple myeloma | 15 | 3 | 20 | |
| Total | 96 | 18 | 19 | (9·7–31·0) |
| Healthy controls | 116 | 2 | 1·7 | (0·1–7·5) |

and with a near-normal SACE (mean 22·0 u/ml, range 14·6–27·6).

We therefore examined the relationship between SACE activity and mortality over 3 and 12 months in non-Hodgkin patients, to determine whether low SACE was related to poor prognosis in general.

The results are shown in Table IV. Regarding the whole series, mean SACE was significantly lower in patients who expired within 3 months than in the surviving patients. Furthermore, in patients with chronic leukaemia and multiple myeloma who died within 3 months and 12 months respectively, all had a significantly lower enzyme activity than survivors.

Amongst all patients with SACE below the normal range (12·0 u/ml) there was a significantly higher mortality after 3 months and one year than in other patients (Table V).

### DISCUSSION

The finding of a low SACE in malignant diseases of blood and lymphatic tissue is in

TABLE IV.—*SACE and survival*

| | | Outcome after 3 months | | | Outcome after one year | | |
|---|---|---|---|---|---|---|---|
| | | Dead | Alive | | Dead | Alive | |
| | (No.) | SACE u/ml ± s.d. (No.) | | $P$* | SACE (u/ml ± s.d.) (No.) | | $P$* |
| Hodgkin's disease | 27 | 18·5 ± 8·1 (3) | 22·3 ± 7·3 (24) | n.s. | 21·2 ± 6·9 (6) | 21·8 ± 7·6 (21) | n.s. |
| Non-Hodgkin lymphoma | 25 | 20·7 ± 5·6 (6) | 20·6 ± 6·0 (19) | n.s. | 22·1 ± 5·3 (10) | 19·5 ± 6·2 (15) | n.s. |
| Acute leukaemia | 14 | 17·9 ± 7·0 (6) | 20·8 ± 6·5 (8) | n.s. | 17·1 ± 6·0 (9) | 22·0 ± 6·9 (5) | n.s. |
| Chronic leukaemia | 15 | 10·2 ± 0·7 (4) | 18·9 ± 5·8 (11) | $P < 0·01$ | 13·4 ± 5·1 (7) | 19·4 ± 6·0 (8) | $P < 0·05$ |
| Multiple myeloma | 15 | 14·1 ± 4·6 (4) | 21·5 ± 4·4 (11) | $P < 0·01$ | 13·5 ± 4·3 (5) | 21·6 ± 4·3 (10) | $P < 0·01$ |
| Total | 96 | 16·7 ± 7·0 (23) | 20·8 ± 6·5 (73) | $P < 0·05$† | 18·2 ± 7·0 (37) | 21·0 ± 6·6 (59) | n.s.† |

* Mann-Whitney rank-sum test. † Student's *t* test.

part agreement with that of Lieberman *et. al.* (1979), who found a slightly decreased SACE in 19 patients with Hodgkin's disease, but reported normal SACE in 11 patients with lymphocytic leukaemia and high SACE in 3/11 patients with Lennerts lymphoma (not represented in the present series).

The results of the present paper contrast with the high SACE reported in sarcoidosis, and they underline the difference between the nature of sarcoidosis and, for example, Hodgkin's disease. In sarcoidosis, the frequency of high SACE has been reported from 29% (Turton *et al.*, 1979) to about 60% (Lieberman *et al.*, 1979) depending on the composition of the series in respect of race, disease activity and duration of disease. Thus our previous results suggest an association between SACE and duration of disease and activity among 85 sarcoidosis patients; 20 patients with active disease for more than 2 years before examination had an SACE of 49·0 u/ml ± 12·7 (s.d.) and high SACE in 85% of the patients, in contrast to 35 patients with sarcoidosis for less than 2 years, where SACE was high in 49%, with a mean of 40·1 u/ml ± 15·9 (Rømer, 1979).

Besides sarcoidosis, only Gaucher's disease has been consistently related to extremely high SACE activity (Lieberman & Beutler, 1976) whereas SACE is decreased in lung cancer (Silverstein *et al.*, 1977; Lieberman *et al.*, 1979) and untreated tuberculosis (Rømer, 1980*a*). For leprosy the results are contradictory (Studdy *et al.*, 1978; Lieberman *et al.*, 1979).

TABLE V.—*Survival of 96 patients with malignant lymphomas, leukaemia and multiple myeloma, related to frequency of SACE* < 12·0 *u/ml*

| | At 3 months | | At 12 months | |
|---|---|---|---|---|
| | Dead | Alive | Dead | Alive |
| SACE < 12·0 u/ml (n = 18) | 10 | 8 | 12 | 6 |
| SACE ⩾ 12·0 u/ml (n = 78) | 13 | 65 | 25 | 53 |
| Total | 23 | 73 | 37 | 59 |
| $\chi^2$ | 6·33 | | 8·66 | |
| $P$ | < 0·05 | | < 0·01 | |

The lack of non-significance of the lower SACE among prednisone-treated patients does not contradict other reports on a generally low SACE in steroid-treated non-sarcoid patients (Turton *et al.*, 1979). The non-significance may be because the "prednisone-treated" patients in the present series included patients who had stopped treatment with prednisone within 2 months.

Furthermore, the results suggest that SACE analysis may be helpful in evaluating patients with mediastinal and hilar masses, and may give valuable information when non-caseating epitheloid granulomas are found.

An explanation for the low SACE in the malignant diseases examined cannot be given at present. The difference in SACE between moderately healthy and extremely ill myeloma patients, and the relationship between SACE level and mortality in chronic leukaemia suggest that perhaps enzyme production is reduced in the terminal stage of disease.

We can conclude that SACE is generally low in the diseases examined, but without a consistent pattern. The greatest clinical value of SACE analysis in this context is as a supplement to other methods of evaluating patients with intrathoracic lymphnode enlargement and in diagnosis and monitoring of patients with sarcoidosis.

This study was supported by the Danish Medical Research Council (Statens Lægevidenskabelige forskningsråd), Grant No. 512-15085. SACE analysis was skillfully performed by laboratory technician Kirsten Golczyk at the Research Laboratory, Department C.

### REFERENCES

BRINCKER, H. & WILBEK, E. (1974) The incidence of malignant tumours in patients with respiratory sarcoidosis. *Br. J. Cancer*, **29**, 247.

CARBONE, P. P., KAPLAN, H. S., MUSSHOF, K., SMITHERS, D. W. & TUBIANA, M. (1971) Report of the Committee on Hodgkin's Disease Staging Classification. *Cancer Res.*, **31**, 1860.

CHASE, M. W. (1966) Delayed-type hypersensitivity and the immunology of Hodgkin's disease, with a parallel examination of sarcoidosis. *Cancer Res.*, **26**, 1097.

CUSHMAN, D. W. & CHEUNG, H. S. (1971) Spectrophotometric assay and the properties of the angiotensin converting enzyme of rabbit lung. *Biochem. Pharmacol.*, **20**, 1637.

DOUGLAS, D. S., DAUGHADY, C. C., SCHMIDT, M. & SILTZBACH, L. E. (1976) Kinetics of monocyte receptor activity for immuno-proteins in patients with sarcoidosis. *Ann. N.Y. Acad. Sci.*, **278**, 190.

GOLDFARB, B. L. & COHEN, S. S. (1970) Coexistent disseminated sarcoidosis and Hodgkin's disease. *J. Am. Med. Ass.*, **211**, 1525.

KITAHARA, N., EYRE, H. J. & HILL, H. R. (1979) Monocyte functional and metabolic activity in malignant and inflammatory diseases. *J. Lab. Clin. Med.*, **93**, 472.

LIEBERMAN, J. (1975) Elevation of angiotensin-converting enzyme level in sarcoidosis. *Am. J. Med.*, **59**, 365.

LIEBERMAN, J. (1976) The specificity and nature of serum-angiotensin-converting enzyme elevations in sarcoidosis. *Ann. N.Y. Acad. Sci.*, **278**, 488.

LIEBERMAN, J. & BEUTLER, E. (1976) Elevated serum angiotensin-converting enzyme in Gaucher's disease. *N. Engl. J. Med.*, **295**, 1142.

LIEBERMAN, J., NOSAL, A. & SCHLESSNER, L. A. (1979) Serum angiotensin converting enzyme for diagnosis and therapeutic evaluation in sarcoidosis. *Am. Rev. Resp. Dis.*, **120**, 329.

NEIMAN, R. S. (1977) Incidence and importance of splenic sarcoid-like granulomas. *Arch. Pathol. Lab. Med.*, **101**, 518.

RØMER, F. K. (1979) Angiotensin-converting enzyme in sarcoidosis. *Acta Med. Scand.*, **206**, 27.

RØMER, F. K. (1980*a*) Angiotensin-converting enzyme as a diagnostic aid in newly diagnosed sarcoidosis compared with cancer of the lung and tuberculosis. (In Danish.) *Ugeskr. Læger*, **142**, 806.

RØMER, F. K. (1980*b*) Sarcoidosis and cancer. A critical view. *Proc. VIIth Int. Conf. Sarcoidosis Other Granulomatous Diseases.* Eds. Williams & Davies. Cardiff: Alpha Omega. p. 567.

SILVERSTEIN, E., FRIEDLAND, J., KITT, M. & LYONS, H. A. (1977) Increased serum angiotensin-converting enzyme activity in sarcoidosis. *Israel J. Med. Sci.*, **13**, 995.

SILVERSTEIN, E., FRIEDLAND, J., LYONS, H. A. & GOURIN, A. (1976) Markedly elevated angiotensin-converting enzyme in lymphnodes containing non-necrotizing granulomas in sarcoidosis. *Proc. Natl Acad. Sci. U.S.A.*, **73**, 2137.

SILVERSTEIN, E., PERTSCHUK, L. P. & FRIEDLAND, J. (1979) Immunofluorescent localization of angiotensin converting enzyme in epithelioid and giant cells of sarcoidosis granulomas. *Proc. Natl Acad. Sci. U.S.A.*, **76**, 6646.

SOFFER, R. L. (1976) Angiotensin-converting enzyme and the regulation of vasoactive peptides. *Ann. Rev. Biochem.*, **45**, 73.

STUDDY, P., BIRD, R., JAMES, D. G. & SHERLOCK, S. (1978) Serum angiotensin-converting enzyme (SACE) in sarcoidosis and other granulomatous disorders. *Lancet*, ii, 1331.

TURTON, C. W. G., GRUNDY, E., FIRTH, G., MITCHELL, D., RIGDEN, B. G. & TURNER-WARWICK, M. (1979) Value of measuring serum angiotensin I converting enzyme and serum lysozyme in the management of sarcoidosis. *Thorax*, **34**, 57.

WHITTAKER, J. A., SLATER, A., AL-ISMAIL, S. A. D., & 4 others (1978) An assessment of laparotomy in the management of patients with Hodgkin's disease. *Quart. J. Med.*, **47**, 291.

YOSHIDA, T., SILTZBACH, L. E., MASIH, N. & COHEN, S. (1979) Serum migration inhibitory activity in patients with sarcoidosis. *Clin. Immunol. Immunopathol.*, **13**, 39.